# A user-centred approach to developing bWell, a mobile app for arm and shoulder exercises after breast cancer treatment

**DOI:** 10.1007/s11764-017-0630-3

**Published:** 2017-07-24

**Authors:** Helena Harder, Patrick Holroyd, Lynn Burkinshaw, Phil Watten, Charles Zammit, Peter R. Harris, Anna Good, Val Jenkins

**Affiliations:** 1 Sussex Health Outcomes Research and Education in Cancer (SHORE-C), Brighton and Sussex Medical School, University of Sussex, Brighton, UK; 2 School of Engineering and Informatics, University of Sussex, Brighton, UK; 3 Brighton and Sussex University Hospitals NHS Trust, Brighton, UK; 4 School of Psychology, University of Sussex, Brighton, UK

**Keywords:** Breast cancer, Shoulder function, Lymphoedema, Exercise, mHealth, Mobile app, Rehabilitation

## Abstract

**Purpose:**

The study aim was to develop a mobile application (app) supported by user preferences to optimise self-management of arm and shoulder exercises for upper-limb dysfunction (ULD) after breast cancer treatment.

**Methods:**

Focus groups with breast cancer patients were held to identify user needs and requirements. Behaviour change techniques were explored by researchers and discussed during the focus groups. Concepts for content were identified by thematic analysis. A rapid review was conducted to inform the exercise programme. Preliminary testing was carried out to obtain user feedback from breast cancer patients who used the app for 8 weeks post surgery.

**Results:**

Breast cancer patients’ experiences with ULD and exercise advice and routines varied widely. They identified and prioritised several app features: tailored information, video demonstrations of the exercises, push notifications, and tracking and progress features. An evidence-based programme was developed with a physiotherapist with progressive exercises for passive and active mobilisation, stretching and strengthening. The exercise demonstration videos were filmed with a breast cancer patient. Early user testing demonstrated ease of use, and clear and motivating app content.

**Conclusions:**

bWell, a novel app for arm and shoulder exercises, was developed by breast cancer patients, health care professionals and academics. Further research is warranted to confirm its clinical effectiveness.

**Implications for cancer survivors:**

Mobile health has great potential to provide patients with information specific to their needs. bWell is a promising way to support breast cancer patients with exercise routines after treatment and may improve future self-management of clinical care.

**Electronic supplementary material:**

The online version of this article (doi:10.1007/s11764-017-0630-3) contains supplementary material, which is available to authorized users.

## Introduction

Axillary treatment for breast cancer, which is surgical removal of the lymph nodes in the armpit (axilla) or axillary radiotherapy, can contribute to upper limb dysfunction (ULD). Symptoms of ULD include pain, numbness, decreased shoulder range of motion (ROM), reduced strength, joint restrictions, axillary web syndrome (lymphatic cording), and development of arm volume changes or lymphoedema due to interruption or damage to the axillary lymphatic system [1–7]. Severity of symptoms and prevalence vary considerably because of differences in diagnostic criteria, assessment methods and time points among studies. However, research data show that half of patients report at least one moderate-to-severe symptom in the first 18 months post surgery [8, 9]. For the majority, symptoms decrease over time, but for some, they are more persistent or develop later [10–14]. More extensive treatment is usually associated with greater morbidity. Common predictors for limitations include mastectomy, axillary lymph node dissection or clearance, higher body mass index and adjuvant therapy (i.e. axillary radiotherapy) [9, 15–20]. ULD is often found to be related to functional limitations in work or social/recreational activities, and psychosocial burdens such as negative affect and reduced quality of life (QoL) [14, 20–27].

Multifactorial physical therapy (e.g. specific passive and active mobilisation or stretching exercises) after breast cancer treatment has proven to be safe and effective [28–31]. Exercises can alleviate symptoms, restore shoulder function and contribute to better QoL, especially if it is implemented early postoperatively [28, 32–35]. In the UK, the National Institute for Health and Clinical Excellence clinical guidelines recommend to provide breast cancer patients with information about the risks of developing lymphoedema and the benefits of exercise [36]. Most patients lack access to an exercise or rehabilitation programme led by a physiotherapist or other health care professional (HCP) [37]. Routine care in many breast cancer centres is information provision and patient education, usually in the form of generic information sheets with exercise instructions or breast cancer charity leaflets or DVDs (e.g. ‘Exercises after breast cancer surgery’ from Breast Cancer Care [38]). However, there appears to be a wide variation in the exercise recommendations for shoulder mobilisation [39, 40], and adherence can be low because it is challenging for many patients to perform the exercises at home without (in-person) training or support [41, 42]. This indicates that there is a need to improve the self-management of postoperative arm and shoulder exercises for breast cancer patients. Self-management refers to the engagement of patients in managing the medical, behavioural and emotional consequences of their long-term disease [43]. It emphasises patients’ control over their disease and is beneficial in terms of patient activation and self-efficacy [44].

In recent years, the widespread adoption and use of mobile technologies is opening new and innovative ways to improve health and health care delivery [45, 46]. A promising way of supporting the delivery of cancer care is the use of mobile health (mHealth) [47]. mHealth can be described as medical or public health practice supported by mobile or other wireless devices [48]. Smartphones and mobile application (apps) have become an integral part of everyday life for an increasing number of people. Seventy-seven percent of all adults in the UK own a smartphone with the average user spending 25 h online each week [49]. There are now around 165,000 health-related apps available for the two major mobile device software platforms: Apple’s iOS (i.e. iPhone Operating System) and Google’s Android [50]. This includes hundreds of cancer-related apps which were designed for several purposes such as increasing awareness, providing information or managing side-effects [51–53].

Although mHealth tools have been suggested as having the potential to impact on supportive care and self-management, most apps are under-used [54, 55]. Only few developers have engaged target users and HCPs in its development to ensure that the content of the app is evidence-based and theoretically informed, and that desired information and features are included to enable early adoption and implementation [51, 52, 56]. The purpose of the present study was to design and develop a mobile app together with target users to support self-management of arm and shoulder exercises following breast cancer treatment. This paper documents the development process of this app (bWell) and preliminary results of early user testing.

## Methods

The study was conducted in two sequential phases. The study objective of phase 1 was to design bWell using a user-centred approach and to build and test the prototype [57]. In phase 2, feedback was collected from a small group of breast cancer patients who used the app post surgery. Study approvals were gained from the National Research Ethics Service (NRES) Committee London—Camberwell St Giles and Brighton (phase 1; REC ref.: 14/LO/1797), NRES Committee North West—Greater Manchester West and Sussex University Hospitals NHS Trust (phase 2: REC ref.: 15/NW/0814) and Sussex University Hospitals NHS Trust. A multidisciplinary team was involved in the study project (including a physiotherapist, a breast surgeon, (health) psychologists and digital media technologists), and regular project meetings were held.

### Focus groups with breast cancer patients

#### Design and setting

A qualitative research design and focus group methodology were chosen to collect user needs and preferences for the content and features of bWell [58, 59]. Focus groups allow for experiences and multiple views to be shared and discussed, and allow for consensus on a topic to be explored [60]. Two serial focus groups with breast cancer patients were conducted at Sussex Health Outcomes Research and Education in Cancer (SHORE-C) at the University of Sussex in Brighton. The focus groups were facilitated by members of the research team who had experience in conducting focus groups or user-led development of mobile apps.

#### Sample and recruitment

Women with early-stage breast cancer (I–IIIA) who completed surgery, were aged 18 years or older and were able to provide written informed consent were eligible for study participation. Participants were recruited between January and March 2015 during follow-up visits in the local breast cancer clinic (Park Centre for Breast Care in Brighton) and through provision of recruitment flyers in the clinic’s waiting room and breast cancer support groups. Those who were interested were given a study information pack and were asked to return the expression of interest (EOI) form with contact details by email or freepost. Potential participants were provided with further study details and screened over the phone to determine study eligibility. Out-of-pocket expenses (i.e. travel by taxi or own transport to attend the focus groups) were reimbursed. All participants were provided with a £25 voucher as thank you gift for the research participation.

#### Data collection

Written informed consent was obtained from participants before commencement of the first focus group. Participants completed a demographic sheet providing background details, including breast cancer diagnosis and treatment, and information on their use of mobile devices. At the start of each focus group, the facilitators (HH, PH) introduced the project, outlined the objectives and highlighted the general ground rules.

Semi-structured topic guides were used to frame the group discussions. Topics of the first focus group (March 2015) included previous experiences of breast cancer treatment (i.e. axillary treatment, ULD), experiences with the arm and shoulder exercises (e.g. information provision, previous experiences) and desired app content and features (e.g. views on background information, images/text exercises). Inclusion and preference for adoption of exercise behaviour change techniques (BCT), such as goal setting, feedback on behaviour, methods for data capture and reminders, were also discussed. A list of potential BCTs for the app were selected by the researchers (PRH, AG, HH) after searching literature and studies that used BCTs to promote self-care or self-management of exercise.

The results of the first discussion were used to develop a wireframe of bWell and mock-up screenshots. Wireframes are basic visual schematics that represent the framework or layout of an app, and are used to develop screen functionality, content layout and priority or sequencing of what needs to happen with the app. Sample screenshots are used in design studies to gather valuable user feedback on overall usability issues (e.g. layout, font size, colour) prior to the final design stage [61, 62]. During the second focus group (June 2015), a series of (PowerPoint) slides illustrating different pages of the app was presented to the whole group and used to encourage opinions and further discussion. Topics included acceptability and attractiveness of features, potential concerns and recommendations for improvements. The facilitator guided participants through each screen, probing for understanding, expectations and areas of confusion. Observations were documented, and in addition, field notes were made by an independent observer. At the end of each session, facilitators reflected their interpretations to the participants to support the credibility of the data interpretation. Each focus group lasted about 1 h.

#### Data analysis

The focus groups were audio recorded and transcribed verbatim with participants’ permission. Descriptive statistics were used to analyse demographic and other quantitative data. All qualitative data was coded and arranged in themes using thematic analysis [63]. The analysis was framed by the core focus group topics. Transcripts were read through several times to identify categories or themes (HH, PH). After initial coding highlighting relevant discussion themes, all text segments were iteratively analysed. Themes were added or merged until they effectively represented all text segments and captured the essence of the discussion [64]. The coding frame was refined with discussions about areas of disagreement and consensus, and any differences in interpretation were reconciled. Comments on user needs and preferences and app usability were classified using the following classifications: content and information (e.g. relevant outcome variables, exercises, functionalities or written information), navigation and structure (e.g. location where information is located) and design and presentation (e.g. use of colour, graphs, amount of text). The study team reached consensus on the content and features that would be included in the app. Considerations for requirement selection (i.e. which features to include) included the number of participants who mentioned it, the context of use, overlap/integration with existing information, technical feasibility and finance and time available to realise the requirement.

### Development of the arm and shoulder exercise programme

An evidence-based exercise programme was developed together with a chartered physiotherapist. A rapid review was conducted to search for (clinical practice) guidelines of arm and shoulder exercises for breast cancer patients [65]. Search terms (e.g. physiotherapy, exercise, rehabilitation, shoulder, upper limb, mastectomy, breast cancer surgery, range of motion/ROM) and resources (e.g. Scopus, EMBASE, MEDLINE, CINAHL, Cochrane database of systematic reviews, Chartered Society of Physiotherapist) were selected, and the results were viewed and summarised by the research team (LB, HH). In addition, information for breast cancer patients (i.e. hospital exercise information sheets, charity leaflets or DVDs) was examined. Both rapid reviews informed the content and structure of the programme. Features of exercise programmes, such as information on initiation (e.g. early or delayed) and progression (e.g. passive vs. active movements, number of stages in the programme), frequency (e.g. number of repetitions and session per day) and range of motion (below or above shoulder level), were extracted and discussed in detail.

### Preliminary early user testing

The feasibility of using bWell after surgery for breast cancer was evaluated in a small group of patients to improve content quality and to evaluate the content interface (visual design) and ease of use. Recruitment took place in the breast cancer clinic via recruitment flyers and/or invitation from a breast cancer surgeon or oncologist. Potential participants registered their interest in the study either via email or an EOI form. They subsequently received a telephone call from the research team to discuss content, study procedures and consent. Selection criteria included the following: newly diagnosed with early-stage breast cancer; treated with surgery (i.e. lumpectomy or mastectomy without immediate reconstruction); being a smartphone user (iPhone model 4–6s); age 18 years or older; ability to provide written informed consent. Participants received a code to download bWell on their mobile device via Apple’s digital distribution platform (detailed instructions were provided via email and a video demonstration on YouTube). Participants were asked to use the app for 8 weeks and were sent a study-specific questionnaire at the end of the study. This questionnaire was developed to capture user feedback and evaluate content and functionality (including ratings) and explored areas of improvement to incorporate in the final design of bWell (Supplementary Fig. III). All participants provided written informed consent prior to start of the study.

## Results

### Focus groups with breast cancer patients

#### Sample

Nine women with early-stage breast cancer participated in the focus groups. Sample characteristics are displayed in Table 1. Participants were between 3 and 18 months post diagnosis, and age ranged from 47 to 65 years. All women had a smartphone or tablet computer, and five used apps on a regular basis. Themes of the focus groups are presented in Table 2 and Supplementary Table I, including typical comments in each category for illustration.

**Table 1:** Sample characteristics focus groups

Socio-demographics characteristics	
Age, mean (SD)	52.3 (5.7)
Education (*n*)	
Primary/secondary school	2
College degree	1
University degree	6
Living arrangements (*n*)	
Alone	6
With partner	3
Occupational status (*n*)	
Working	7
Not working	2
Health characteristics	
Months since diagnosis, mean (SD)	12.1 (6.3)
Type of treatment (*n*)	
Surgery only	2
Surgery, adjuvant treatment^a^	7
Mobile device user experience	
Mobile device use^b^ (daily average), *n*	
30–60 min	5
1–2 h	2
>2 h	2
Use of mobile device features (*n*)	
Text messaging	9
Apps	5
Internet browsing	6
Camera	8
Games	2
Other^c^	4

^a^Six of nine received adjuvant treatment at time of study (radiotherapy 2, Herceptin 1, tamoxifen 3)

^b^All patients used a mobile phone, and seven of nine used a tablet computer

^c^Other: alarm, email, social media

**Table 2:** Results focus group: experiences with ULD and the arm and shoulder exercises

Emerging themes	Representative quotes
Awareness of importance of exercises	• *I can’t remember if I was told at discharge* [about exercises] *… but it was in the pack and I just read everything in there because my movement is really crucial to my job and my livelihood, so I was scared stiff of not being able to move properly. I started them straight away and yes they are painful, but one year on they have made a difference.* [Participant 6] • *I went a few steps ahead. I had breast conserving surgery and moved on, thought ‘I’m fine’ until I was driving and had to get into tight spaces. I didn’t realise how much it was affecting me. It’s hard to measure it.* [Participant 5]
Awareness that exercises are ongoing	• *It’s an ongoing exercise, to do them all the time, to keep them going* [Participant 2] • *Initially I was really good* [doing the exercises] *they were all impressed. I was moving on and it was only 4 or 5 months later, I was doing something and thought ‘oh the pain’ … and only since then have I started to do the exercises again and it is a lot better.* [Participant 1]
Lacking or inconsistent advice	• *I wasn’t told about it … I knew about it because a friend had been diagnosed before and she lent me a DVD she had been given 3 years ago. The BCN didn’t specifically mention it, and I’m quite surprised because she was thorough in any other way* [Participant 7] • *I had the BCN and surgeon saying ‘Start the day after* [surgery]*’, but then the district nurse visited and said ‘You only just had surgery, don’t worry about it.’ Then I saw my oncologist … He was concerned about cording and advised to go swimming. Two days later I saw the surgeon and BCN, and they said ‘Absolutely no way that you should go swimming!’ … I was terribly upset and felt that I failed.* [Participant 4]
Gaps in care pathway and follow-up	• *Nobody asked anything about it at all. It was very much here you go do your things* [Participant 8] • *I’m about to start chemotherapy and my nurse said ‘Well that’s it, I won’t be seeing you for a while’… There are different BCNs, no regular appointments. The focus is on chemo now and not my arm* [Participant 4]
Need for more directions or physiotherapy	• *I remember just getting through it and not speaking to anyone* [about exercises] *I had to get through it and just do it* [Participant 2] • *I was really surprised that there was no intervention from the physio. I thought that the two would be really well connected, but there’s no connection with physio at all!* [Participant 3]

*BCN* breast care nurse, *ULD* upper limb dysfunction

#### Experiences with arm and shoulder exercises

Experiences with axillary treatment and arm and shoulder exercises varied widely (see Table 2). Most patients (7/9) were informed about the exercises by a HCP (surgeon or breast care nurse), but some indicated that the information and instructions they received was partial and felt they needed more direction. Others commented on the inconsistency among HCPs regarding management of ULD and showed dissatisfaction with the advice they received about the exercises, and care and use of their affected arm. Some were confused or frustrated by this conflicting information. Most agreed that the lack of consistent advice was related to the treatment trajectory such as being monitored by different HCPs (surgeon, oncologist) in a relatively short time.

A key finding was that all women were aware of the importance of the exercises for their recovery and performed them daily in the first weeks post surgery. Some were surprised about the intensity of ULD symptoms and how it affected their everyday life. Most realised that the exercises are ongoing and have to be continued long after treatment, sometimes because of fear of developing (recurrent) ULD symptoms. Suggestions for information on how to manage expectations towards exercises, and reassurance about what can be expected after treatment, were recommended by participants.

#### User needs and requirements for bWell

A detailed overview of the functionalities to improve rehabilitation after axillary treatment is displayed in detail in Supplementary Table I. All participants expected that bWell would be of clinical benefit for breast cancer patients, and most commented that a mobile app would have supported them after treatment. Some proposed app features were more prominent; in particular, the use of reminders or push notifications to trigger exercise sessions emerged several times in the discussion and was viewed as very useful for the app. Participants also referred to a need for tailored information on ULD and the arm and shoulder exercises, and thought that this would promote recovery. There was a strong preference for video demonstrations (i.e. instruction videos of each individual exercises, and a longer video for each stage with the exercises performed in a sequence) including verbal instructions to help patients understand and safely perform the exercises at home. Some suggested adding a timer function to the exercises and a possibility to repeat them. Most women highlighted that it was important the videos would provide reassurance about abilities, and motivate patients.

Participants valued functions for feedback and self-monitoring and mentioned that for example a tracking feature to self-rate well-being (emotional, physical) and a visual display (e.g. a personal graph) would be encouraging, increase awareness and be helpful to see change over time. They stated that the timing and frequency of the reminders needed to be well designed so that they could be customised by the users. The majority of participants expressed concerns about using goal setting features. Some remarked that if predetermined outcome goals were not achieved, this could have a negative impact on motivation and emotional status. A function for system reward (e.g. stars) for user engagement was mentioned by one participant. Some women also considered that features for sharing personal information (i.e. feature that enables users to communicate with each other, receive/ask for tips, advice or encouragement from other patients, such as a chat feature, comment area or ‘like’ option) could be beneficial and motivating in certain circumstances.

#### Feedback on wireframe and screenshots

The second focus group with six of nine participants centred on feedback on wireframes and mock-up sample screenshots for bWell to validate usability and explore design ideas. Initial feedback on wireframes and screenshots was very positive and helped to clarify some design concepts. Participants commented on the self-monitoring feature (i.e. scales for pain, flexibility and mood) and suggested to rephrase the mood scale and colour-code the ratings. All agreed that one rating a day would be sufficient to keep a record of progress. They also recommended the inclusion of a diary function for self-evaluation of symptoms (e.g. a notes feature in which users can create/edit and store notes) so they could register events for certain days on the calendar. It was suggested that a brief section with tailored information (e.g. a list of common problems) and a FAQs section would benefit users. Participants commented on an exercise demonstration video and recommended that the model should have an ‘everyday-look’ (e.g. plain clothes, jewellery). Also a choice of models of different ages to engage a wider range of patients and help them to relate or identify with the model was proposed. Suggestions from the second focus group were discussed by the research team and used to generate the final user needs and system requirements for bWell.

### Development of the arm and shoulder exercise programme

#### Framework for the exercise programme

A rapid review of relevant literature and guidelines identified several programmes for arm and shoulder mobilisation after breast cancer treatment but also showed a wide variability regarding content and structure of the exercises [28, 30, 31, 66]. There was no consensus on intervention duration, frequency of training, training volume (number of sets and repetitions) and progression of exercises to more advanced performance levels. Information on strategies to facilitate adherence was lacking. Most studies recommended early initiation of exercises (i.e. within 1–3 days post surgery) to avoid deterioration of full shoulder ROM, and a delayed approach to full mobilisation (i.e. limited movement of the arm below 90° up till 7–10 days post surgery) [28, 34, 66–70]. Data also showed growing support for the use of progressive resistance training and weight training at a later stage as both were considered safe and efficacious in breast cancer rehabilitation [29, 33, 71–73]. Based on a rapid evidence assessment, an exercise programme for bWell was developed which incorporated three key stages of exercise progression with the aim to maintain/increase ROM, facilitate lymphatic transport and increase strength and stability of the affected arm and shoulder:

- Stage 1: early post-operative exercises for passive mobilisation (six active assisted exercises below shoulder level) starting the first day after surgery
- Stage 2: intermediate post-operative exercises for active mobilisation and stretching (four exercises above shoulder level) starting after 1 week after surgery or when the drains are removed
- Stage 3: late post-operative exercises for muscle strengthening and sustained stretching (five exercises) starting 4–6 weeks after surgery depending on wound healing and recovery

The exercises were chosen for their ability to be performed independently without special equipment. They included mobilisation in all planes of motion, including flexion, extension, adduction, abduction and internal/external rotation. Stage 3 included exercises with weights (or similar household objects such as cans or small water bottles) and a series of Pilates exercises which were demonstrated to be effective and safe in studies with breast cancer patients [74–76]. All sessions were initiated with warm-up exercises, and cool-down exercises were recommended at the end of each session. Three sets of five repetitions (stages 1 and 2) or two sets of 10 repetitions (stage 3) were recommended prior to moving on to the following exercise. Each exercise stage would take approximately 15 min to complete. In line with other programmes, the bWell exercises were not suitable for patients who had immediate breast reconstruction.

#### Filming the exercise demonstration videos

Videos of the arm and shoulder exercises were produced by the Media Technology Lab at the University of Sussex and filmed with a multicamera setup against a solid white background (Supplementary Fig. I). These videos were used as visual demonstration of how to perform each exercise (17 in total including a warm-up and cool-down exercise) and to help patients understand the exercises for each post-operative stage. Exercises for each stage were also filmed in a sequence to provide a class to follow along. The exercises were demonstrated by a female volunteer who had breast cancer surgery 9 months before filming. All videos were scripted by the researchers and reviewed by a breast cancer patient and adjusted accordingly. Language for film scripts and content text was adapted to be clear and concise by use of short sentences, subheadings and highlighted text. Film script included reminders about exercising within the pain-free range and without discomfort, and to maintain good body alignment for core stabilisation. Text to reassure patients about exercise level and ability was also added. The content of the videos was checked by the physiotherapist to assure that the exercises were performed correctly. All videos were edited and included in bWell, together with text-based material for each exercise and stage (see Table 3 and screenshots in Supplementary Fig. II).

**Table 3:** bWell content and behaviour change techniques

Feature content	Description	Behaviour change technique^a^
Information provision	Information about ULD, the recovery stages and all individual exercises based on evidence-based guidelines	Education/information
Video demonstrations	Detailed video instructions of each exercise and each recovery stage	Demonstration of behaviour
Graded tasks	Progressively more difficult exercises to increase arm/shoulder ROM and strength	Education/information
Record exercises performed	Calendar feature to record if each session of exercises was performed	Monitor and record behaviour
Motivational statements	Verbal instructions (including messages for reassurance) for each exercise	Motivation
Push notifications, including opt-in opt-out option	Self-setting reminder function to prompt patient daily to access next exercise session to increase engagement and user retention	Motivation
Behaviour and progress tracking	Self-rate pain, flexibility and mood each day that the app is used with visual display in calendar to see change over time	Self-monitoring of outcome/feedback on outcome of behaviour
Diary function	Diary function in calendar to allow reflection on daily function	Self-monitoring of behaviour
Section FAQs	Questions about the purpose of bWell, e.g. technical issues, emails, non-technical user questions	Education/information
Web links	Web links to contact information about breast cancer related resources or support	Education/information

*ULD* upper limb dysfunction

^a^BCTs as described and classified by Michie et al. [77]

### Building bWell

After evaluation of the wireframes and mock-up screenshots, alterations to the functional specifications and user interface design of bWell were made in accordance with the recommendations. Technical requirements were derived, after which the first prototype was developed (see Table 3 and Supplementary Fig. II). bWell was built using Swift programming language with the Xcode IDE [78, 79]. Quality control testing was carried out by the developers (PH, PW) throughout the development process to ensure that all content and features were functioning properly. bWell version 1.0 was submitted to a digital distribution platform (App Store) via an iOS Developer Account.

### Preliminary early user testing

Between January and March 2016, four breast cancer patients contacted the research team and provided consent for early testing. One woman had technical problems with downloading the app on her iPad and withdrew from the study. Participants were aged between 51 and 58 years, had completed surgery (i.e. lumpectomy and sentinel node biopsy) and were scheduled for radiation treatment. All used bWell on an iPhone 5, almost daily or several times per day, in preparation for their radiotherapy. They found navigation easy and reported no technical issues. Content text and font size were clear and concise. Popular characteristics were the demonstration videos and reminder feature. Participants stated that the videos (including instructions) were very useful and clear. Self-monitoring and feedback on outcomes (self-rating and visual graphs) were viewed or seen as somewhat useful. Open-ended feedback indicated that especially the graded tasks and different stages of the exercise programme were beneficial for recovery from treatment. All women reported that the app kept them motivated and engaged, and would definitely recommend bWell to other breast cancer patients (average star rating: 4.6/5).

## Discussion

This paper presents a user-centred approach and co-creation process of bWell, a mHealth app for breast cancer patients. The importance of engaging users in the design and conduct of mHealth interventions is well recognised as it facilitates app adoption and usage [80, 81]. This project is one of few to involve patients in the development process and a first step in optimising self-management of ULD in patients with breast cancer. ULD affects a great number of survivors, and specific exercises are recommended to restore full shoulder mobility and minimise symptoms or development of lymphoedema. bWell is a theory-informed mobile app that supports self-management of these exercise routines. The use of self-management programmes has been promoted to reduce symptoms, achieve better health-related outcomes and reduce the demand on health services and health costs [82, 83].

During the first phase of our study, we evaluated women’s own experiences with breast cancer treatment and ULD. Results from the focus group showed that patients were aware of the importance of arm and shoulder exercises, but advice from HCPs was often inconsistent or sometimes lacking. Similar findings were observed by Lee et al. who examined perceptions of arm care and exercise advice in 175 breast cancer patients, 6 to 15 months after their surgery [84]. Women revealed concerns about ULD or fear of lymphoedema, and reported that standardised advice did not meet expectations.

Our study also identified barriers and motivators for adoption and continued use of an arm and shoulder exercise app in breast cancer patients. The top desired features among focus group participants were reminders and detailed video demonstrations of the exercises. Tailored information about ULD and tracking exercise and progress were also perceived to be helpful behavioural change features for bWell. Goal setting features were seen as a barrier in the uptake of the app. Results of early user testing showed that it is feasible to use the app after breast cancer surgery. Women commented on the easy navigation and clear content, and reported that they would definitely recommend the app to other patients.

The current study has some limitations. Study participants were self-selected and may have been more likely to be interested in the use of mobile technology. However, the focus groups included non-users of mobile apps which expanded the diversity of the group. We were also unable to include all user needs and requirements identified in the focus groups (e.g. social support function, motion capture device) in the current version of the app because of financial limitations. Lastly, early testing was conducted in a small sample of users. This was due to the limited time for this pilot project (12 months in total) which restricted recruitment, and because bWell is currently only available (as a free app without in-app purchases) for iOS devices which was a reason for non-participation for some women. Future work should include further development of bWell, such as including other mobile platforms (e.g. Android) and adding an online forum for social support, and collecting feedback about bWell from a large group of HCPs. Further research should also engage patients without mobile devices for example by providing them with a smartphone or tablet for the duration of the study.

## Conclusions

bWell, a novel mobile app, was developed together with breast cancer patients to support self-management of arm and shoulder exercises following axillary treatment. Further evaluation in a randomised controlled trial is needed to provide objective evidence on its clinical effectiveness. This will allow us to optimise the usability of the app and to examine if it is feasible to use bWell as an integrated part of clinical care for breast cancer.

## Electronic supplementary material

Supplementary Table I (DOCX 26 kb)

Supplementary Figure I (DOCX 597 kb)

Supplementary Figure II (DOCX 1100 kb)

Supplementary Figure III (PDF 347 kb)
