# Supplementary material for: A user-centred approach to developing bWell, a mobile app for arm and shoulder exercises after breast cancer treatment
Source: J Cancer Surviv. 2017 Jul 24;11(6):732–42. doi: 10.1007/s11764-017-0630-3 (PMC5671540; doi:10.1007/s11764-017-0630-3)
Supplement: Supplementary file 1 — (DOCX 26 kb) [file 11764_2017_630_MOESM1_ESM.docx]

**Supplementary material - Table I: Results Focus Group: mobile app requirements and features**

| **Mobile app features (+)** | **Representative quotes** |
| --- | --- |
| Video demonstration and verbal instructions (+) | - *I think it is important that the exercises are demonstrated, therefore you got to have a visual element as well as someone speaking to you, that’s important* [Participant 7] - *Quite honestly seeing a video with someone doing it rather than a flat picture would have helped enormously. The words* [in leaflets] *are quite ambiguous; don’t fit the image very much … a short video of each* [exercise] *so you can look at it, and in a class. If someone did the exercises with you … It could be your own personal coach* [Participant 8] |
| Tailored information (+) | - *If you could have an app that would have that information on there, so week 1 what to do, that manages your expectations and tells you what you can and can’t do?* [Participant 4] - *There could be buttons tailored to different circumstances. Something like ‘post-surgery 1-7days’, ‘post-surgery with drain’, … it might reassure someone* [Participant 5] |
| Push notifications (+) | - *I think notifications in terms of time would be really important. So not just on screen but if it was a buzz or a musical note, an alarm to remind people that it’s time to do it again* [Participant 8] - *So it could be that you did it* [the exercises] *and then it reminded you that you still got to do it so many times* [Participant 4] |
| Timer on exercises (+)^a^ | - *For some moves it should be interested to know how long to do them. Have a timer on it. You should be holding it for a minute, or holding at a stretch point.* [Participant 5] - *You could have a timer showing how long you should be doing the exercise, a timer that ticks down* [Participant 3] |
| Goal setting | - *There’re problems with goals because they are different for everybody* [Participant 4] - *Maybe some older women could be quite put off by that, it needs to be encouraging. If they’re doing anything at all it’s good, especially at first. It* [the app] *just has to encourage people to do it* [Participant 5] - *If you didn’t reach them* [your goals] *you would feel a bit down* [Participant 1] |
| Track exercise performance (+) | - *Like a school teacher, tick you’ve done it, and it will tell you to do the next one* [exercise] [Participant 7] - *There should be a checklist with how many times you should do it and the recommended times that you should be doing it … You can log in each day and see what you’ve done, it gives you feedback* [Participant 6] |
| Progress tracker, including graphs (+) | - *That’s* [graph] *really good because when you’re in it every day, it’s really easy to forget that last week you couldn’t lift your arm and that you can see I can do it now, I’m obviously making progress!* [Participant 4] - *Your personal graph, it’s encouraging* [Participant 2] |
| Reassurance about abilities and progress (+) | - *But you can have someone saying ‘Don’t worry if you’re meeting it this time, you can work towards it* [Participant 6] - *It’s helpful to be told what to expect and how you should feel* [Participant 7] |
| Motion capture device for movement analysis and feedback | - *I thought I was doing really well with the exercises until I stood in front of the mirror… Then I realised that it [affected arm] wasn’t quite doing what it should be doing, so it* [an app] *should be… that should be part of it, that you actually look at yourself* [Participant 4] - *So if you were able to have a trajectory measure of someone holding the phone in their hand and doing the exercises and you would be able to provide feedback on whether someone … made it from here to here, for example* [Participant 8] |
| Real life stories | - *That idea about everybody stories, you could have a real story, ‘This is my life and I did do the exercises’* [Participant 4] |
| Social support function | - *It could be a social possibility. If there was a social forum so that women who are going through the same thing, that they can support each other … have reassurance from peers if you’re having a bad day. If the app could support that, I think that would be really, really good* [Participant 8] |
| Endorsement HCP | - *I wondered if the app could include some sort of video with a breast care specialist and perhaps past patients endorsing the exercise program and the importance of it* [Participant 5] |

(+) = included in bWell; HCP = health care professional

^a^ Timing of the exercises (i.e. how long to hold a stretch, number of repetitions) was included in the film script
