# Supplementary figures and images for: A user-centred approach to developing bWell, a mobile app for arm and shoulder exercises after breast cancer treatment
Source: J Cancer Surviv. 2017 Jul 24;11(6):732–42. doi: 10.1007/s11764-017-0630-3 (PMC5671540; doi:10.1007/s11764-017-0630-3)

**Supplementary material - Figure I: Filming the exercise demonstration videos**


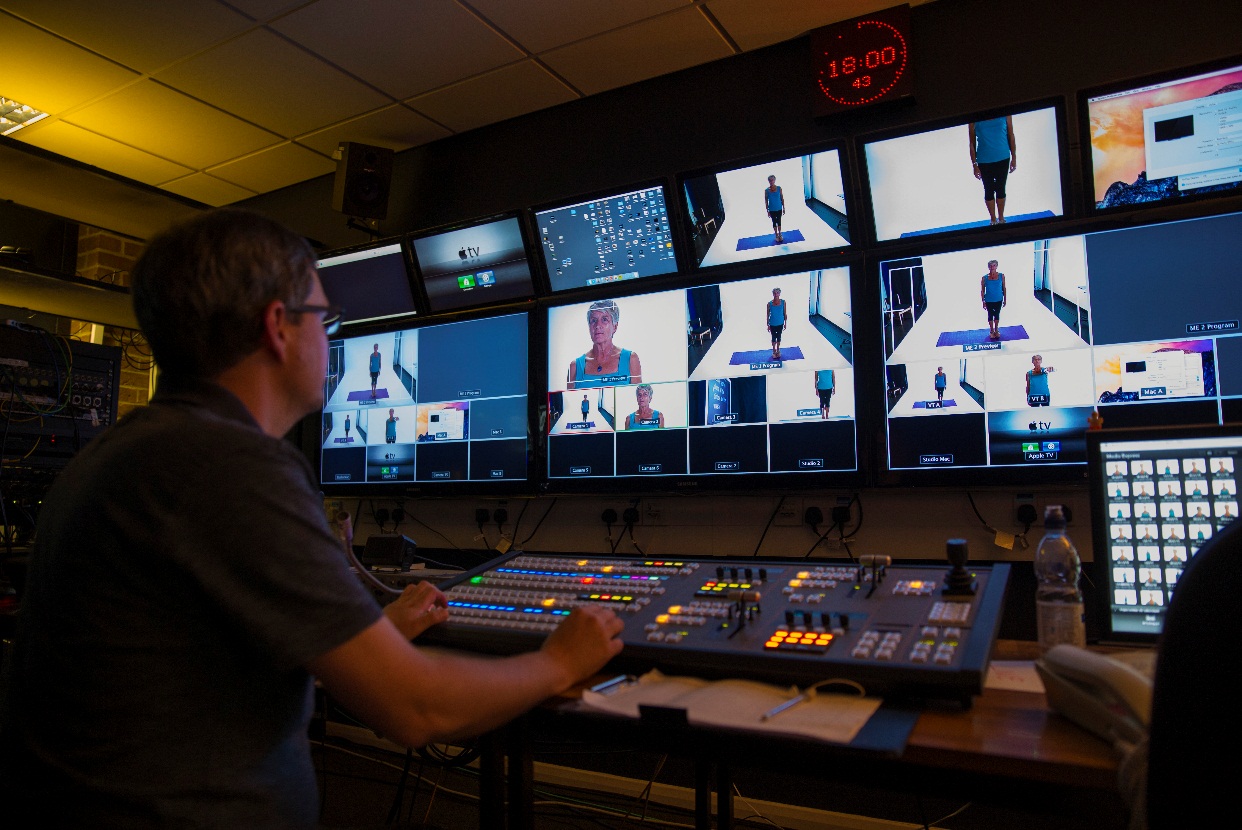


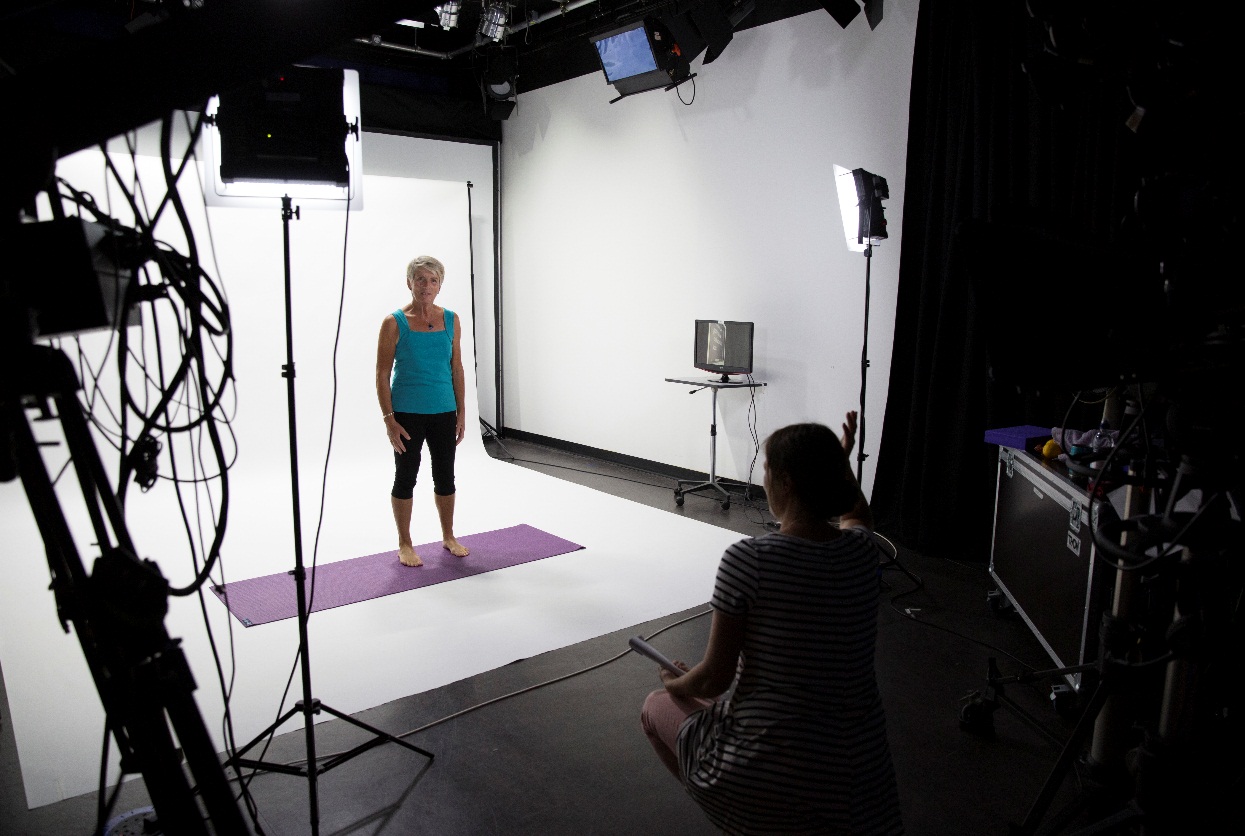

Supplement: Supplementary file 2 — (DOCX 597 kb) [file 11764_2017_630_MOESM2_ESM.docx]
