# Supplementary material for: A user-centred approach to developing bWell, a mobile app for arm and shoulder exercises after breast cancer treatment
Source: J Cancer Surviv. 2017 Jul 24;11(6):732–42. doi: 10.1007/s11764-017-0630-3 (PMC5671540; doi:10.1007/s11764-017-0630-3)
Supplement: Supplementary file 3 — (DOCX 1100 kb) [file 11764_2017_630_MOESM3_ESM.docx]

**Supplementary material - Figure II: Overview of bWell**

**A.**
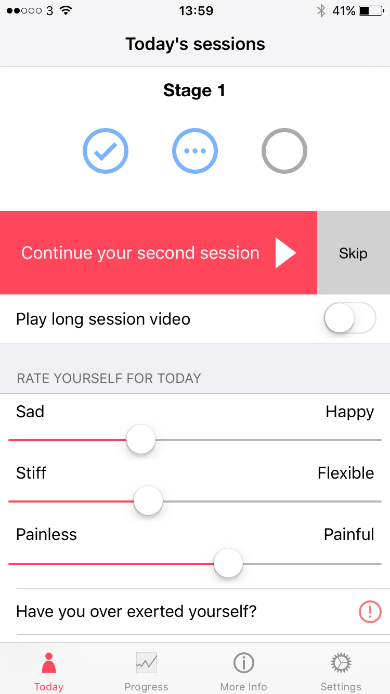
 **B.**
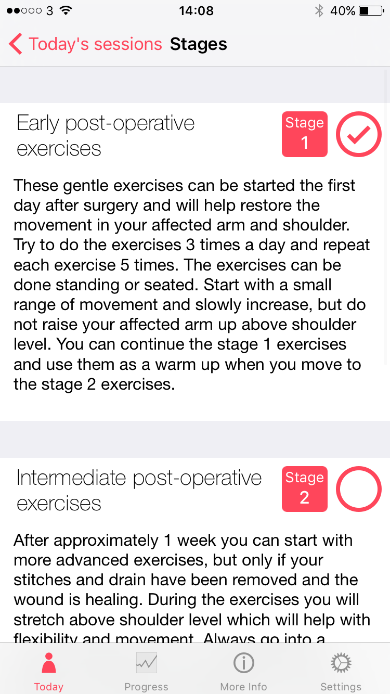


**C.**
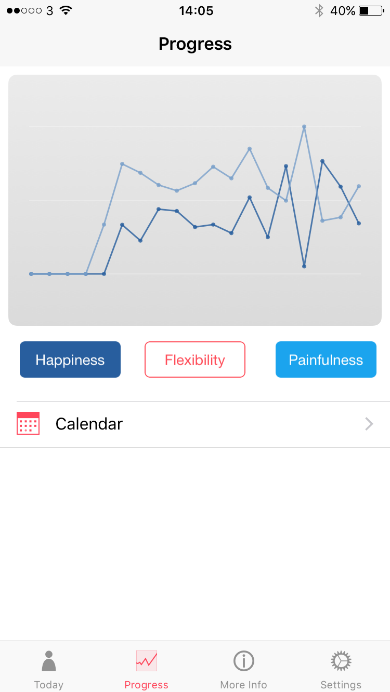
 **D.**
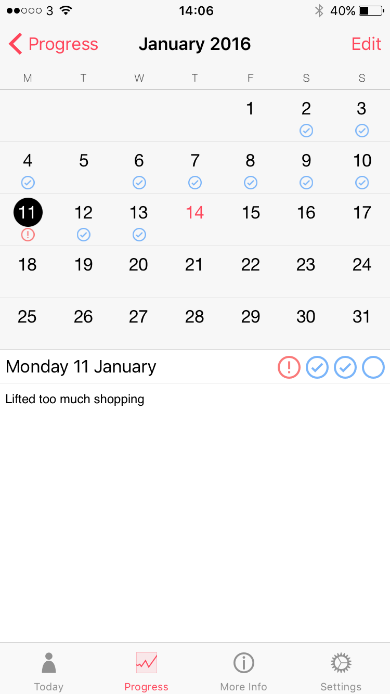
 **E.**
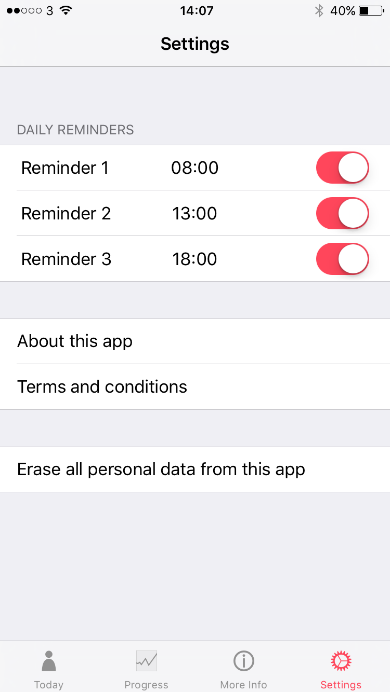


**Supplementary material - Figure II: Overview of bWell - continued**

**F.**
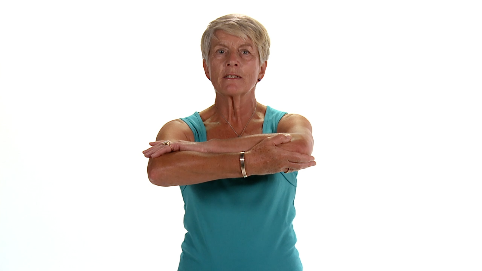
 **F.**
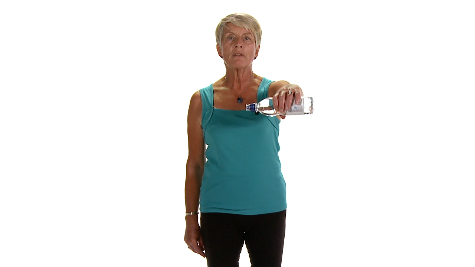


**F.
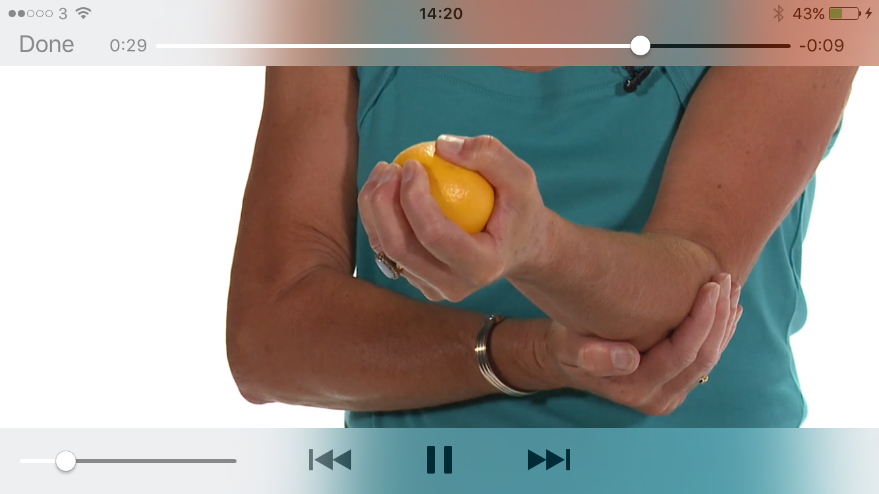
**

**A: start page bWell**

**B: exercise stage selection page**

**C: progress tracking**

**D: calendar and note function**

**E: reminder settings**

**F: exercise images/videos**
