# Supplementary material for: A user-centred approach to developing bWell, a mobile app for arm and shoulder exercises after breast cancer treatment
Source: J Cancer Surviv. 2017 Jul 24;11(6):732–42. doi: 10.1007/s11764-017-0630-3 (PMC5671540; doi:10.1007/s11764-017-0630-3)
Supplement: Supplementary file 4 — (PDF 347 kb) [file 11764_2017_630_MOESM4_ESM.pdf]

Supplementary material – Figure III: App Evaluation Questionnaire

Study ID:
Date:

We would like your views on the exercise app. All feedback will be helpful and treated in the strictest confidence. Could you please rate the different aspects of the exercise app in this questionnaire (please circle as appropriate).

Section A – General information

Which device did you use the app on?

1. iPhone – *please specify model:*	iPhone 4S	iPhone 5<br>Phone 5S<br>iPhone 5C	iPhone 6<br>iPhone 6 Plus
2. iPad – *please specify model:*	iPad 2<br>iPad 3<br>iPad 4	iPad Air	iPad Mini<br>iPad Mini 2
3. iPod Touch (5G model)			

How easy was it to install the app on your device?

1.Very easy 2.Fairly easy 3.Rather difficult 4.Difficult

On average, how often did you use the app during the study?

1. Several times a day 2. Almost daily 3. Few times a week 4. Few times a month

Section B – App design and usability

The app was reliable and there were no technical issues

1. Strongly agree 2. Agree 3. Disagree 4. Strongly disagree

It was difficult to learn how to use the app

1. Strongly agree 2. Agree 3. Disagree 4. Strongly disagree

It was easy to navigate through the app (e.g. moving between the screens)

1. Strongly agree 2. Agree 3. Disagree 4. Strongly disagree

The text for labels and buttons was clear and concise

1. Strongly agree 2. Agree 3. Disagree 4. Strongly disagree

The design of the app was too overwhelming (e.g. too many links or features)

1. Strongly agree 2. Agree 3. Disagree 4. Strongly disagree

The app looked very appealing and enhanced my experience

1. Strongly agree 2. Agree 3. Disagree 4. Strongly disagree

The colours used provided poor contrast and poor readability

1. Strongly agree 2. Agree 3. Disagree 4. Strongly disagree

The font size and spacing of text ensured good readability

1. Strongly agree 2. Agree 3. Disagree 4. Strongly disagree

The app kept me highly motivated and engaged throughout my use of it

1. Strongly agree 2. Agree 3. Disagree 4. Strongly disagree

Supplementary material – Figure III: App Evaluation Questionnaire – continued

Section C – Content

How useful were the videos of the exercises (individual and long session)?
1. Very useful 2. Somewhat useful 3. Useful 4.Not useful at all

How clear were the videos (images)?
1. Very clear 2. Somewhat clear 3. Clear 4.Not clear at all

How clear was the sound?
1. Very clear 2. Somewhat clear 3. Clear 4.Not clear at all

How confusing were the instructions of each video?
1. Very confusing 2. Confusing 3. Somewhat confusing 4.Not confusing at all

How useful was it to have reminders for the exercises?
1. Very useful 2. Somewhat useful 3. Useful 4.Not useful at all

How useful was it to do the daily ratings (mood, pain, flexibility)?
1. Very useful 2. Somewhat useful 3. Useful 4.Not useful at all

How useful were the charts of the daily ratings?
1. Very useful 2. Somewhat useful 3. Useful 4.Not useful at all

Section D – Overall Rating

How helpful did you find the app after surgery?
1. Not at all helpful 2. Somewhat helpful 3.Helpful 4.Very useful

Would you recommend this app to other women with breast cancer?
1. Would not recommend 2. Would maybe recommend 3. Would definitely recommend

What is your overall star rating of the app?
1. * One of the worst apps I've used
2. **
3. *** Average
4. ****
5. ***** One of the best apps I've used

Please write any other comments below

………………………………………………………………………………………………
………………………………………………………………………………………………
………………………………………………………………………………………………
………………………………………………………………………………………………
………………………………………………………………………………………………
………………………………………………………………………………………………
………………………………………………………………………………………………

Thank you very much for completing the questionnaire.
Please return it to SHORE-C in the stamped and addressed envelope.
